# Multi-ROI Spectral Approach for the Continuous Remote Cardio-Respiratory Monitoring from Mobile Device Built-In Cameras

**DOI:** 10.3390/s22072539

**Published:** 2022-03-25

**Authors:** Nunzia Molinaro, Emiliano Schena, Sergio Silvestri, Carlo Massaroni

**Affiliations:** Unit of Measurements and Biomedical Instrumentation, Departmental Faculty of Engineering, Università Campus Bio-Medico di Roma, 00128 Rome, Italy; n.molinaro@unicampus.it (N.M.); e.schena@unicampus.it (E.S.); s.silvestri@unicampus.it (S.S.)

**Keywords:** remote monitoring, smartphone’s built-in camera, heart rate estimation, respiratory rate estimation, multi-ROI approach, unobtrusive monitoring, cardiorespiratory, continuous monitoring

## Abstract

Heart rate (HR) and respiratory rate (*f_R_*) can be estimated by processing videos framing the upper body and face regions without any physical contact with the subject. This paper proposed a technique for continuously monitoring HR and *f_R_* via a multi-ROI approach based on the spectral analysis of RGB video frames recorded with a mobile device (i.e., a smartphone’s camera). The respiratory signal was estimated by the motion of the chest, whereas the cardiac signal was retrieved from the pulsatile activity at the level of right and left cheeks and forehead. Videos were recorded from 18 healthy volunteers in four sessions with different user-camera distances (i.e., 0.5 m and 1.0 m) and illumination conditions (i.e., natural and artificial light). For HR estimation, three approaches were investigated based on single or multi-ROI approaches. A commercially available multiparametric device was used to record reference respiratory signals and electrocardiogram (ECG). The results demonstrated that the multi-ROI approach outperforms the single-ROI approach providing temporal trends of both the vital parameters comparable to those provided by the reference, with a mean absolute error (MAE) consistently below 1 breaths·min^−1^ for *f_R_* in all the scenarios, and a MAE between 0.7 bpm and 6 bpm for HR estimation, whose values increase at higher distances.

## 1. Introduction

The monitoring of vital signs such as the respiratory rate (*f_R_*), heart rate (HR), body temperature, and blood pressure is essential to assess general health status [1]. Among others, HR is an essential indicator. Heart function is affected by many factors such as psychosocial stress, smoking, excessive use of alcohol, malnutrition, lack of physical activity, and congenital diseases [2]. Even *f_R_* plays a vital role in assessing the health status of a subject, providing information on physical deterioration, and in the prediction of cardiac arrest. Moreover, it is related to various stressors (e.g., emotional stress, cognitive load) [3]. Therefore, information about HR and *f_R_* can be used in a wide range of applications such as medical diagnostics, fitness assessment, and mental stress analysis. Most traditional methods used to measure HR and *f_R_* require physical contact with the subject, which represents the main limitation in their usage (e.g., electrocardiography via skin-attached electrodes for HR measure, flow sensors for *f_R_* measurement).

The use of non-contact methods may extend the capability of monitoring health status of users directly in the home environment, thus making possible early diagnosis of disease or the continuous tracking of chronic ones in a less constrained setting outside hospital clinics [4]. Furthermore, with the outbreak of the COVID-19 pandemic, the need to monitor patients remotely to avoid the overload of hospitals as well as to develop telemedicine services has become increasingly assertive. Among the wide range of existing non-contact technologies, such as radar [5] and thermal cameras [6], the adequate processing of videos recorded with mobile devices built-in cameras may be advantageous because of easy accessibility and easiness of use of these devices (e.g., laptops, smartphones).

Estimating HR and *f_R_* from a video recorded with RGB cameras can be performed through color-intensity-based and motion-based methods. Color-based methods are related to detecting subtle skin color changes due to the movement of the blood from the heart to the head which occurs during the cardiac cycle. During the video record of a subject’s face, RGB sensors can detect the remote photoplethysmographic signal (r-PPG signal) related to volumetric changes of blood in facial capillaries [7]. Both HR and *f_R_* can be estimated from the r-PPG signal, as the respiratory activity modulates the cardiac activity [8]. When the r-PPG technique is used to retrieve information about the pulsatile activity through a video recording of a subject’s face, different factors should be considered, including the user–camera distance [9,10], the lighting source [11,12], the resolution of the video acquisition system [13], and the region of interest (ROI) [14,15]. Among these factors, user–camera distance, the lighting source, and the selected ROI can affect the quality of the r-PPG waveform and thus the accuracy in the estimation of HR [9,11,16,17]. The ROI selection for HR estimation is a debated issue, single or multiple ROIs in the facial region [18,19] or the palm hand [20] can be employed, but performances are typically dependent on the specific application and recording environment [18,19]. Conversely, motion-based methods rely on the detection of small amplitude movements recorded by a video camera, such as chest wall movements due to the breathing activity or mechanical movement of the head caused by the flow of the blood from the heart to the head (called remote ballistocardiography—r-BCG) [21,22]. Given the difficulty of isolating r-BCG signals from other movements, HR estimation is very challenging to implement in this way [23]. Conversely, motion-based methods are promising for *f_R_* estimation, especially when the breathing activity is retrieved from the measurement of chest wall movements [24,25] through the optical flow algorithm proposed in [21] to overcome concerns caused by ambient light variations.

Available methods based on both color-based and motion-based methods have some drawbacks and limitations. In most cases, the measurements are taken in a structured environment with expensive cameras that are not easily available and strongly limit the fields of digital cameras’ use for vital signs monitoring. Additionally, the reliable estimation of HR and *f_R_* simultaneously is uncommon, mainly because only r-PPG signal does not provide a robust measure of *f_R_*, and motion-related methods are inaccurate for estimating HR. Additionally, the majority of studies focus on the estimation of only average values or complicated time-domain analysis unaddressable without supervision or with data recorded in real-world settings [12,26,27,28]. In this context, average values are typically investigated in the frequency domain by analyzing the spectrum of the whole signal to obtain only a single value of HR and *f_R_*. This discourages the application of this technology when abrupt increases or decreases in HR and *f_R_* are expected (i.e., sports field and athlete recovery), or when more data are needed even for short-term recordings. 

To tackle these drawbacks, in this paper, we propose a novel approach based on the use of images captured with a mobile device using a multi-ROI approach and spectral analysis for the continuous monitoring of HR and *f_R_* parameters. The method is based upon (i) the automatic detection of multiple landmarks on the face and the torso, (ii) the implementation of seven different post-processing techniques to retrieve both the pulsatile signal based on the changes of optical properties of the facial skin for HR estimation and torso movements for *f_R_* calculation, and (iii) the identification of most informative ROIs for continuously estimating HR and *f_R_* values with an update time of 1 s. As for influencing factors, we considered two recording user–camera distances and the presence or not of an external illumination source, without any constrains on users’ clothing.

## 2. Measuring System: Description and Working Principle

The proposed non-contact measuring system is composed of a hardware module (i.e., built-in camera) for video recording and an algorithm with a twofold aim: (i) preprocessing of the video to obtain the pulsatile and the breathing-related signals, and (ii) post-processing the raw signals for HR and *f_R_* estimation.

The working principle behind the extraction of the pulsatile and breathing-related signals from a video is explained in the following section.

### 2.1. Remote Photoplethysmography: Principle of Work

Remote photoplethysmography (r-PPG) is a technique for remote measurement of human cardiac activities by detecting subtle color variations visible on the subject’s skin utilizing a multi-wavelength RGB camera [29]. r-PPG shares the same principle as PPG, which involves the measurement of light transmitted through or reflected from the skin, capturing the changes in blood volume [30]. The optical properties of human skin are determined by the presence of different chromophores in the layers of the skin. However, skin color is mainly given by the presence of melanin in the epidermis and the hematic pigments (i.e., hemoglobin, oxyhemoglobin, beta carotene, and bilirubin) present in the dermis/hypodermis vascular plexus. Among the hematic pigments, the primary source of rhythmic variation in light absorbance and reflectance is hemoglobin present in blood vessels [31]. When the light falls on the skin surface, the variation of the intensity of reflected light due to the pulse wave activity can be captured by a camera. 

The observed intensity depends on the camera and light source distance to the measurement point in the subject’s skin. Subtle changes can be observed over time in the color depending on the blood circulation, movement, and specular variation in the body.

In recent years, researchers have proposed several r-PPG post-processing techniques for extracting the pulse signal from videos, including blind source separation (BSS) [32,33], model-based [13,34,35], and deep-learning methods [36,37]. 

### 2.2. Hardware for Video Data Recording

The measuring system used in this work consists of a built-in digital smartphone camera (iPhone 6s, Apple Inc., Cupertino, CA, USA) with a resolution of 1280 × 720 pixels with a framerate of 30 fps. The camera is an 8 bits per channel device. The images in RGB space are characterized by the property that each color can be represented using the superposition of three values (i.e., one for each channel), which encode the intensities of red (R), green (G), and blue (B), contributing to the specific color. Since the smartphone camera used in this work has automatic white balance (AWB) functions that allow the variation of the camera’s exposure parameters during recording, to avoid AWB, the videos were recorded using the ProMovie Recorder App (by Panda Apps Limited), which allows locking the exposure parameters at the beginning of the acquisition and keeping them fixed for the whole duration of the video. The shutter and ISO parameters were set up at the start of the acquisition.

### 2.3. Algorithm for the Video Preprocessing

The preprocessing of the recorded video was performed offline via a custom-made algorithm developed in MATLAB environment.

#### 2.3.1. Identification of Regions of Interest (ROIs) on Face and Torso

Firstly, a video including the face and torso regions is recorded using the built-in camera. The pit-of-the-neck and the face are identified in the image automatically via the Viola–Jones algorithm [38]. The ROI at the level of the torso is created from the pit of the neck to retrieve the chest wall movements associated with the respiratory activity [39]. Then, the face is automatically identified, and three ROIs are detected in the first video frame from the face: the right (hereinafter RCheek) and left cheeks (LCheek) and the forehead (FHead) since they are the facial regions with good vascularization [40]. Face ROIs were square shape and size equal to 5% of the frame height for video recorded at 0.5 m and 3% for video recorded at a distance of 1 m.

#### 2.3.2. Respiratory Signal from the ROI Torso

**Figure 1:**
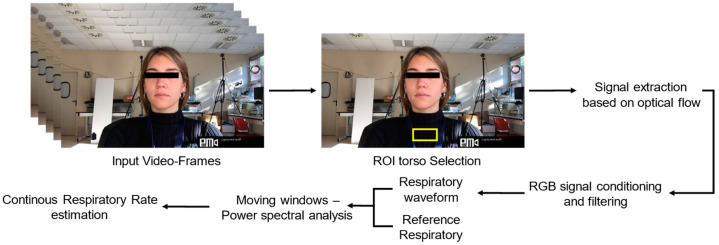
Flowchart presenting the steps carried out to extract the breathing waveform from the ROI torso for continuous estimation of respiratory frequency.

The respiratory signal is extracted from the pit-of-the-neck ROI using a method that relies on the computation of optical flow (hereinafter OF), which allows the estimation of the displacement between two consecutive images by tracking the features of the images [41,42]. Figure 1 reports the proposed framework for the extraction of breathing waveform and the continuous estimation of *f_R_*.

A prior transformation from images in RGB space to greyscale images is required to apply the OF to video frames. In this paper, the Horn and Schunck (HS) algorithm is used to extract OF from the frames [43]. The algorithm assumes that pixels maintain their intensity along their trajectory. Assuming a constant brightness, the intensity of a pixel *I (x, y, f)* at the frame *f* will remain stable for short time and small movements.

For a single frame step *df*, the following equation is valid:

(1) I(x,y,f)=I(x+dx,y+dy,f+df)

where *dx* and *dy* denote the displacements in *x* and *y* directions. Assuming that the pixel displacement is sufficiently small, Equation (2) is obtained:

(2) $$\frac{\partial I}{\partial x}v_{x}+\frac{\partial I}{\partial y}v_{y}+\frac{\partial I}{\partial f}=0$$

where *v_x_* and *v_y_* are the components of the pixel velocity along the *x*-axes and the *y*-axes of the OF of the *I (x, y, f)* that must be determined.

The HS algorithm computes the displacement between two consecutive images by tracking the image features on a pixel-by-pixel basis. In this way, a velocity vector for each pixel in the image is obtained. According to [43], the velocity component along the *y*-axis (i.e., *v(y,f)*) was assumed as the most related to movements of the ribcage caused by breathing. The HS optical flow was applied to all video frames, and the values of the y-velocity vectors within the ROI were averaged to obtain a single value per frame. The average velocity vector (*v_y_*) was integrated to obtain the linear displacement of the rib cage related to the respiratory activity (*s_y_*).

##### 2.3.3. r-PPG Signal from the ROIs Face

**Figure 2:**
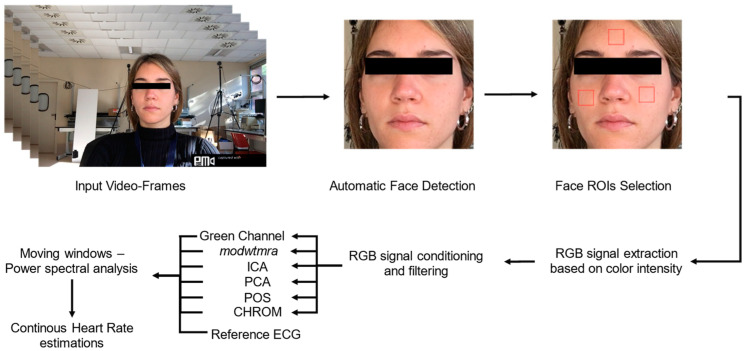
Flowchart presents the steps carried out to extract the r-PPG signals from image sequences for continuous heart rate estimation.

Three time-dependent RGB signals are obtained by spatially averaging the intensity of the pixels within the ROI for each frame. In this way, the raw signals for each ROI are obtained:

(3) $$x_{RCheek}\left(i\right)=\left[x_{R}\left(i\right);x_{G}\left(i\right);x_{B}\left(i\right)\right]$$

(4) $$x_{LCheek}\left(i\right)=\left[x_{R}\left(i\right);x_{G}\left(i\right);x_{B}\left(i\right)\right]$$

(5) $$x_{FHead}\left(i\right)=\left[x_{R}\left(i\right);x_{G}\left(i\right);x_{B}\left(i\right)\right]$$

with *i* = 1,..., N, where N is the number of frames. We then proceed with the conditioning of the raw signals, which involves two steps: (i) raw r-PPG signals detrending [18,44,45] and (ii) smoothness prior approach (SPA) [10,15,46,47,48,49]. At this point, for each ROI, we obtain a three-dimensional signal in time, where the three RGB channels represent the three dimensions. To proceed with a spectral analysis of the signals for HR extraction, it is necessary to reduce the dimensionality of the signal to obtain a one-dimensional signal as a function of time. The choice of which method to use is one of the most debated issues in the r-PPG literature. In this paper, six post-processing techniques have been implemented and compared. They can be divided into color-based methods (i.e., Green Channel Analysis, *modwtmra*, Chrominance-based signal processing method, Plane Orthogonal to Skin) and Blind Source Separation Analysis (i.e., Independent Component Analysis, Principal Component Analysis). The framework of the proposed r-PPG based HR extraction method is as shown in Figure 2.

###### Green Channel Analysis

The green channel analysis is the most used method to derive plethysmographic signals from the analysis of RGB camera recorded videos [14,18,50,51,52]. As demonstrated in the study proposed by Verkruysse and colleagues [53] in 2008, the green channel offers the best signal quality in terms of signal-to-noise ratio (SNR) and accuracy in HR estimation, whereas red and blue are close to the noise value. The signal obtained with this technique is hereinafter reported as r-PPG_Green_.

###### Modwtmra

The *modwtmra* is an algorithm that can be used to enhance the quality of the r-PPG signal obtained from the green channel [54]. The raw signal was filtered using a wavelet symlet filter of the fourth order with decomposition level 4 assumed, allowing the extraction of the signals originating from the heart [54]. The signal obtained with this technique is hereinafter reported as r-PPG_MODW_.

###### Chrominance-Based Signal Processing Method (CHROM)

The chrominance–based signal processing method (CHROM) was first introduced by De Haan and Jeanne [35] to enhance the robustness in the estimation of r-PPG signal. The CHROM technique removes components unrelated to the r-PPG signal by projecting RGB channels into a chrominance subspace. The r-PPG signal is obtained from the difference between two chrominance signals. It will be reported as r-PPG_CHROM_.

###### Plane Orthogonal to Skin (POS)

Plane Orthogonal to Skin uses a different projection orthogonal to the skin tone than the CHROM method, and it is considered more robust in complex illumination scenarios [34]. As in the CHROM method, the r-PPG signal (hereinafter reported as r-PPG_POS_) is obtained from the linear combination of two projection axes giving in-phase signals.

###### Blind Source Separation Analysis

A technique often used for noise removal from physiological signals is Blind Source Separation (BSS), which consists of reconstructing source signals from a set of observed signals. Typically, observations are acquired from the output of an array of sensors, where each sensor receives a different combination of the source signals.

There are several methods of BSS, including Independent Component Analysis (ICA) and Principal Component Analysis (PCA).

ICA technique was first used in the field of remote photoplethysmography by Poh et al. [32] in 2010. The ICA aims to decompose a linear combination of sources under the assumptions of independence and non-Gaussianity [55,56] to estimate the source signal. There are several algorithms to perform ICA, but the JADE ICA algorithm [57] is the most widely used in the field of remote photoplethysmography [9,14,15,32,33,47,48]. The signals obtained with this technique are hereinafter reported as r-PPG_ICA1_, r-PPG_ICA2_ and r-PPG_ICA3_ as results of the first, second, and third ICA estimations.

Similar to ICA, the PCA technique allows the identification of the original source signals (*s_1_(t)*, *s_2_(t)*, and *s_3_(t)*) from a set of observed signals (*x_1_(t)*, *x_2_(t),* and *x_3_(t)*) by recovering the directions along which the data have maximum variance. Its goal is to represent these data as a set of new orthogonal variables named *principal components* [58]. The signals obtained with PCA are hereinafter reported as r-PPG_PCA1_, r-PPG_PCA2_, and r-PPG_PCA3_ as results of the first, second, and third PCA estimations, respectively.

Since ICA and PCA return the independent and principal components randomly, the component whose power spectrum contains the highest peak was selected for further analysis [47].

## 3. Tests and Experimental Trials

### Participants and Tests

In this study, eighteen volunteers (i.e., 15 males, 3 females, age range 20–39 years old, height 163–177 cm, body mass 55–83 kg) were enrolled. Videos were recorded in a 30 m^2^ unstructured environment (i.e., a laboratory) equipped with three windows that provide natural light. All experiments were carried out indoors and with a stable amount of light delivered by neon lights. No restrictions have been placed on the clothing of the subjects.

**Figure 3:**
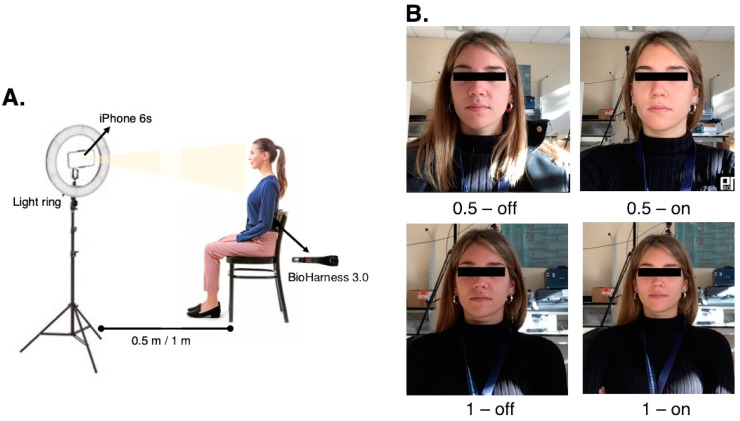
(**A**) Schematic representation of the experimental setup. (**B**) Picture of a subject during the recording of the video in the different experimental conditions (see Table 1 for the operating conditions).

Each volunteer was called to perform four trials while seated and breathing quietly. As reported in Table 1, we investigated two different user–camera distances (0.5 m and 1.0 m) and two illumination conditions (natural light or artificial light), which resemble circumstances that can be found in the home, office, and clinical settings. A light ring (with a diameter of 48 cm and a total power of 55 W) was used to provide the additional (artificial) light source (set light temperature 3500 K). The light ring was installed on a tripod. On the same tripod, the smartphone used for capturing videos was positioned (Figure 3).

A multi-parameter wearable device, the Zephyr BioModule BioHarness 3 by Medtronic (BH3), was used to record the reference respiratory and electrocardiographic (ECG) signal contextually to the video recording. This system consists of a thoracic belt and an electronic module. It acquires the user’s breathing pattern by sensing the volumetric changes in the thorax employing a strain gauge and the ECG waveform via dry electrodes. The reference breathing and ECG signals were sampled at 25 Hz and 250 Hz, respectively.

The protocol used was the same for the four trials: subjects at the beginning of each trial performed a full-lung apnea lasting ~5 s to allow synchronization between the data acquired by the reference system and those acquired by the video recording system. After the apnea, subjects continued to breathe quietly until the end of the test. Subjects were asked not to wear glasses, as their presence has been shown to affect r-PPG signal detection negatively [18]. In addition, all subjects were asked not to wear make-up. At the beginning of each trial, the environmental light intensity values of the scenario were measured using a light meter (see Table 1).

Four videos—of approximately 90 s each—were recorded for each subject under different conditions. All the tests were carried out in compliance with the Ethical Approvals (ST-UCBM 27/18 OSS), and prior to the tests, all the participants provided their informed consent.

## 4. Data Analysis

**Figure 4:**
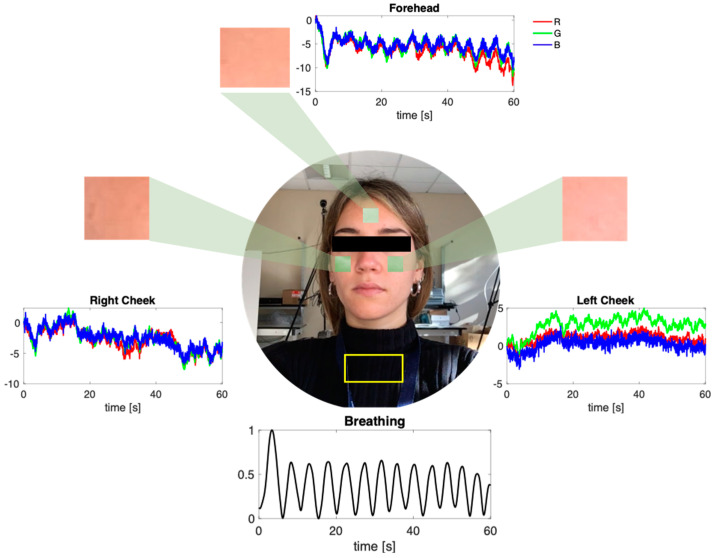
Example of raw r-PPG signals extracted from the three ROIs and the breathing signal extracted by the OF technique from the ROI torso.

The collected videos were post-processed in MATLAB environment to extract both the breathing patterns and the r-PPG signals from the described post-processing techniques (Figure 4). We retrieved the *s_y_* signal from each trial by processing the optical flow method and the r-PPG signals with the six implemented post-processing techniques used to retrieve the cardiac pulsatile waveform. Before data processing, all the r-PPG signals were filtered in the range 0.5 Hz–2.5 Hz (equivalent to 30–150 bpm range) to emphasize the pulsatile component caused by the heart beating. Even the respiratory signals obtained from the video were filtered with a first-order Butterworth bandpass filter between 0.05 Hz and 2 Hz to remove the high frequencies related to noise but preserve all the possible *f_R_* values (range 3–120 breaths·min^−1^).

All the r-PPG signals obtained from the video were synchronized with the ECG waveform by using the respiratory traces recorded from the video (*s_y_*) and the reference breathing signal from BH3. The endpoint of the apnea was used as a synchronization point on both the *s_y_* and the reference system signal. The first 60 s of the video were considered for the analysis.

After this process, the analysis in the spectral domain was carried out to estimate the values of HR and *f_R_* both for reference and video signals. The Power Spectrum Density (PSD) was computed using the Lomb periodogram through a moving window of 20 s [51], sliding by 1 s. The window length was selected according to [51], in which windows with sizes between 15 s and 20 s were found to represent a good deal between time resolution and noise robustness.

The values of HR and *f_R_* were computed by considering the frequency at which occurs the highest peak of the *PSD* in each window, according to the following equation:

(6) $$HR\;\left[bpm\right]=\frac{60}{\frac{1}{f_{max}^{PSD}}}\;$$

(7) $$f_{R}\;\left[breaths\cdot min^{-1}\right]=\frac{60}{\frac{1}{f_{max}^{PSD}}}$$

**Figure 5:**
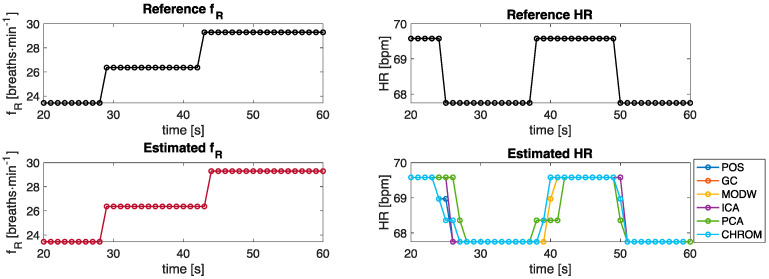
Temporal trends of estimated *f_R_* and HR values against the reference values.

**Figure 6:**
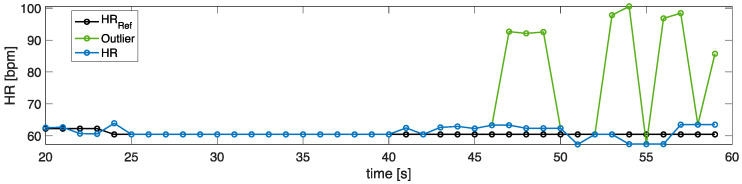
Example of HR values obtained from the windowing analysis. In black, the reference HR values, in green the outlier values; in blue, the estimated HR values after the outlier removal and their replacement.

Figure 5 shows the temporal trends of the estimated values of *f_R_* and HR against the reference values. Sometimes, some estimated HR values are inconsistent with the other values since r-PPG signals are affected by artifacts. These values are defined as outliers (Figure 6). Considering the total vector of estimated values, those falling outside the interquartile range multiplied by 1.5 were removed and replaced with the nearest non-outlier values.

Three multi-ROI approaches were proposed for the HR estimation:

(1) Single-ROI approach: HR values were separately estimated from the r-PPG signals extracted from the three identified ROIs (i.e., LCheek, RCheek, and FHead) per each post-processing technique.
(2) Multi-ROI approach: HR values were estimated by averaging the HR values gathered from all the ROIs as the following equation:

(8) $$HR_{mean}=\frac{HR_{LCheek}+HR_{FHead}+HR_{RCheek}}{3}\;$$

where *HR_LCheek_*, *HR_FHead_*, and *HR_RCheek_* are the HR values estimated by using the PSD analysis in each window.

(3) SNR-based approach: per each post-processing technique, the signal that better represents the pulsatile waveform was identified by evaluating the signal-to-noise ratio (i.e., SNR) according to [35]. Only the signal with the highest SNR value was used for HR estimation.

We used Bland–Altman and correlation analysis (coefficient of determination R^2^) to investigate the agreement between the implemented approaches and the reference values. Per each approach (i.e., single-ROI, multi-ROI, SNR-based), we carried out a separate analysis to evaluate the influence of different ambient conditions on HR and *f_R_* estimation. Firstly, we used the data collected at two different distances (i.e., 0.5 m and 1.0 m), using the data collected in two different lighting conditions. Then, we evaluated the influence of the two lighting conditions (i.e., natural and artificial light) using the data collected at 0.5 m and 1.0 m.

Additionally, for the multi-ROI and the SNR-based approaches, the Mean Absolute Error (MAE) and the Root Mean Square Error (RMSE) were computed.

## 5. Results

### 5.1. Respiratory Rate Estimation

**Figure 7:**
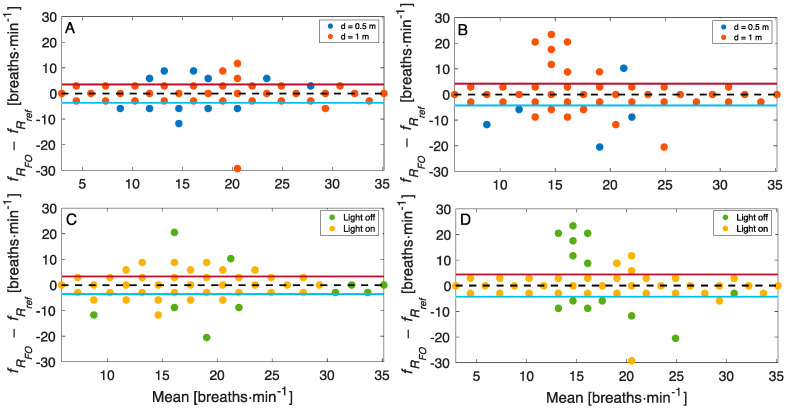
Bland–Altman plots comparing *f_R_* estimated through optical flow in different ambient conditions: (**A**) when the light ring is on; (**B**) when there is only natural light; (**C**) at a distance of 0.5 m; (**D**) at a distance of 1.0 m.

Figure 7 reports the Bland–Altman plots for the *f_R_* values estimated through OF to evaluate the influence of the user–camera distance (Figure 7A,B) and the influence of two lighting conditions (Figure 7C,D). The dashed line represents the Mean of Difference (MOD), and the red and blue lines represent the upper Limit of Agreement (LOA) and the low LOA, respectively. A MOD ± LOAs of −0.05 ± 3.58 breaths·min^−1^ is obtained when the light ring is on considering both the data collected at the distance of 0.5 m and 1.0 m, and a MOD ± LOAs of −0.02 ± 4.22 breaths·min^−1^ is achieved in the case of natural light (Figure 7B). Considering all the data collected in the two lighting conditions, a MOD ± LOAs of −0.13 ± 3.43 breaths·min^−1^ and a MOD ± LOAs of 0.06 ± 4.34 breaths·min^−1^ were obtained at distances of 0.5 m and 1.0 m, respectively.

In Table 2, MAE and RMSE values are reported per each trial by considering all the subjects’ data. Independently of the user–camera distance and the lighting conditions, MAE is always below 1 breaths·min^−1^, and the higher RMSE is obtained at a distance of 1.0 m when the light ring is off. 

### 5.2. Heart Rate Estimation

The influence of user–camera distance and lighting conditions in the estimation of HR was investigated by analyzing LOAs values obtained from Bland–Altman plots, R^2^, MAE, and RMSE. These values were computed per each implemented post-processing technique and for the three approaches followed for HR estimation (i.e., single-ROI, multi-ROI, and SNR-based approaches).

#### 5.2.1. Single-ROI Approach

Table 3 reports the values of MOD ± LOAs obtained per each implemented post-processing technique and for the three identified ROIs, as well as the results obtained with the multi-ROI approach (see the following section). In the majority of cases, light-off trials showed higher LOAs. The same was true in the trials with a user–camera distance of 1 m, independently of light conditions. The single-ROI approach does not allow the identification of the best region of the face for HR estimation because of conflicting results in the presence/absence of light at the two distances (first two columns) and between the two distances considering both the lighting conditions (third and fourth columns).

**Figure 8:**
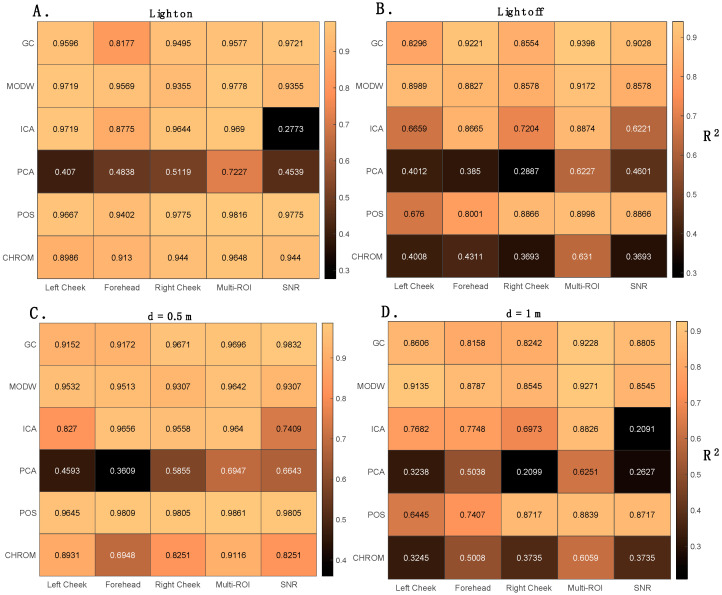
Correlation analysis between the HR values estimated with the implemented post-processing techniques and the reference system in different conditions: (**A**) when the light ring is on; (**B**) when there is only natural light; (**C**) at a distance of 0.5 m; (**D**) at a distance of 1.0 m. The darkest color represents the minimum value of R^2^, whereas the lightest color is the highest value of R^2^.

Figure 8 shows the values of R^2^ between the HR values estimated with the three approaches (i.e., single-ROI, multi-ROI, and SNR-based approach) and the reference values per each implemented post-processing technique. The R^2^ corroborate the results obtained with LOAs. Except for PCA (showing lower R^2^ values in all the conditions), R^2^ ranged from 0.8177 to 0.9775 in light on conditions and are lower when the light is off for all the ROIs. At a user–camera distance of 1 m, the R^2^ values are generally lower than at 0.5 m.

#### 5.2.2. Multi-ROI Approach

**Figure 9:**
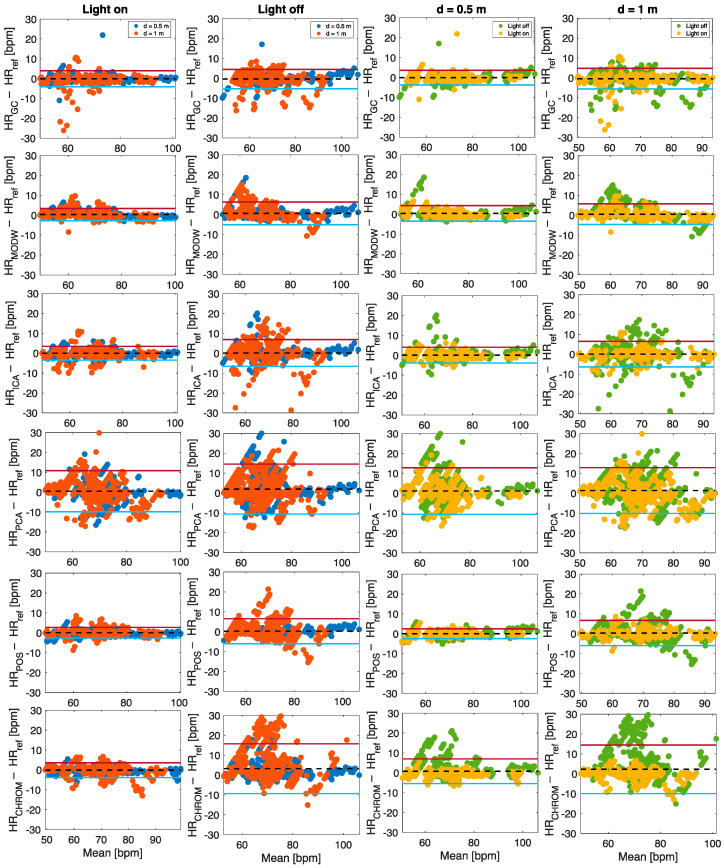
Bland–Altman plots comparing the average HR estimated by using six different post-processing techniques in different ambient conditions.

As anticipated, Table 3 reports the results related to the HR estimation using the multi-ROI approach. Considering the average value of HR estimated with this approach, all the implemented techniques show good agreement in the different ambient conditions, and the best agreement is achieved from the POS algorithm. To evaluate the influence of lighting condition in the estimation of average HR, all data collected at the two distances were used (*n* = 1476, considering that 41 values were obtained per volunteer). In all the cases (light on and off, d = 0.5 m and d = 1 m but expect for the ICA), the performances achieved with the multi-ROI approach outperform those provided by the single-ROI approach (see Figure 8 and Table 3). From Figure 9 (*n* = 1476 per Bland–Altman plot), when the light ring is on, the HR estimations are more accurate when compared against the reference ones. All the implemented post-processing techniques show better performances at 0.5 m (higher R^2^ and lower LOAs), and the POS algorithm allows obtaining the best agreement with reference values (MOD ± LOAs = 0.009 ± 2.45 bpm, R^2^ = 0.9861). Compared to all the techniques, PCA was the worst.

Table 4 reports the values of MAE and RMSE in bpm computed per each implemented post-processing technique in the estimation of average HR at different ambient conditions. Considering all the post-processing techniques, MAE is between 0.7 bpm and 6 bpm, and RMSE is in the range 1.2 bpm to 9.11 bpm. The minimum value of MAE is achieved under the conditions 0.5 m of distance and light on for the POS algorithm (MAE = 0.65 bpm), whereas the minimum value of RMSE is obtained under the conditions 0.5 m of distance and light off for POS algorithm (RMSE = 1.2 bpm). The values of MAE and RMSE increase at a distance of 1.0 m when the light ring is off, and the maximum values are obtained for the CHROM algorithm (MAE = 5.91 bpm and RMSE = 9.11 bpm).

#### 5.2.3. SNR-Based Approach

**Figure 10:**
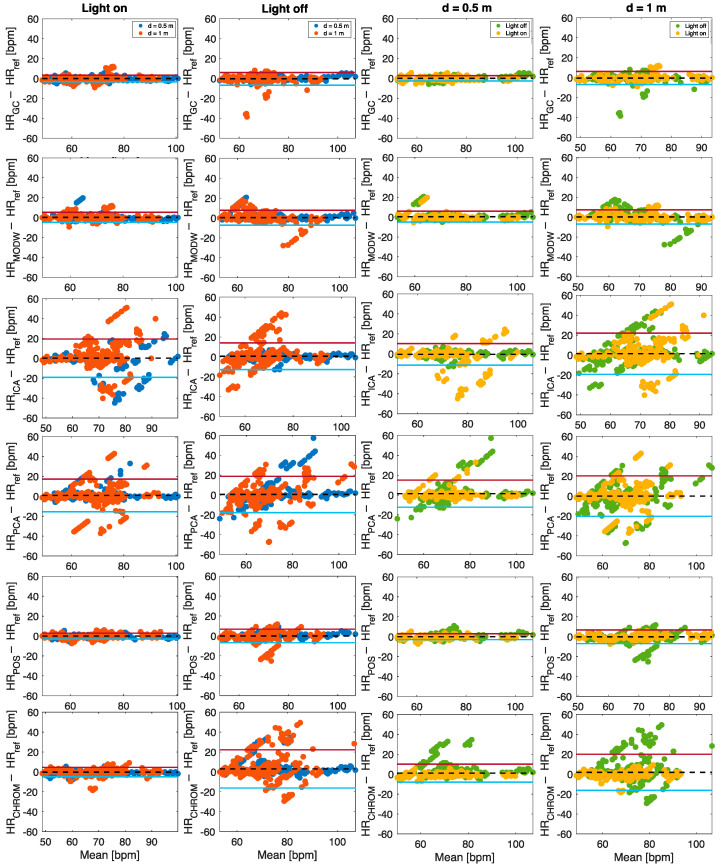
Bland–Altman plots comparing HR estimated from the r-PPG signals with the SNR-based approach.

Considering the R^2^ provided in Figure 8, the SNR-based approach estimations present lower correlation with the reference data compared to the multi-ROI approach. In line with the results obtained with the multi-ROI approach, HR values estimated at 0.5 m are closer to the reference values than those estimated at 1 m. Light-on data collections provided more accurate HR values estimations for all the post-processing techniques (Figure 10). The values of MOD ± LOAs obtained in the estimation of HR from r-PPG signals with the highest SNR value per each implemented post-processing technique are reported in Table 5.

Data show that the POS algorithm allows obtaining the best agreement with reference values (MOD ± LOAs = 0.05 ± 2.91 bpm, R^2^ = 0.9805), slightly higher than the best one found with multi-ROI approach. POS and Green Channel techniques presented close results in terms of MOD and LOAs for all the conditions as emerge from the bolded values in Table 5. 

MAE and RMSE values strengthen this result (see Table 6). The values of MAE are in the range 0.60 bpm and 6.71 bpm, which are achieved for the Green Channel at 0.5—off and for CHROM at 1—off, respectively. Similarly, the lowest RMSE value is obtained for the Green Channel technique (RMSE = 1.27 bpm), and the highest is achieved for the CHROM (RMSE = 12.98 bpm). At high user–camera distance, the values of MAE and RMSE increase for all the implemented post-processing techniques except for *modwtmra,* which shows an MAE of 1.10 bpm at 0.5—on and an MAE of 0.84 bpm at 1—on. The values of MAE and RMSE increase at 1.0—off, and the maximum value is obtained for the CHROM algorithm (MAE = 6.71 bpm and RMSE = 12.98 bpm).

## 6. Discussion

In this paper, a novel approach for the continuous estimation of HR and *f_R_* based on a multi-ROI approach and spectral analysis of RGB video-frames recorded with a mobile device is proposed. A built-in smartphone camera is used to record videos of the subject’s face and torso, allowing the recording of chest wall movements and subtle color changes related to blood volume pulse in a non-intrusive and low-cost manner. The respiratory signal is estimated by the motion of the chest, whereas the cardiac signal is from the pulsatile activity at the level of right and left cheeks and forehead. We have investigated the performance in scenarios simulating daily living to evaluate the influence of environmental factors as recording distance (i.e., 0.5, 1.0 m) and illumination conditions (natural or artificial light). We implemented and tested several commonly adopted post-processing techniques on video frames to (i) estimate HR and *f_R_* values through single, multi-ROI, and SNR-based approaches; (ii) carry out a continuous estimation of HR and *f_R_* values with an update time of 1 s; and (iii) investigate the influence of user–camera distance and light source on the performances of the implemented approaches used to obtain HR and *f_R_* values. 

Considering all the trials, we obtained comparable MAE and RMSE values in the estimation of *f_R_* with the OF technique applied on the single torso ROI. Independently of the user–camera distance and the lighting conditions, MAE values are always below 1 breaths·min^−1^, which is in accordance with the results obtained in [50], in which breath-by-breath *f_R_* values were computed through an analysis in the time domain. Considering all data collected in the two illumination conditions, at 0.5 m, LOAs of ± 3.43 breaths·min^−1^ are achieved, which are slightly worse than the results obtained in [43], in which LOAs of ± 1.92 breaths·min^−1^ were obtained for breath-by-breath *f_R_* values computed with an analysis in the time domain, which required the identification of inspiratory peaks and appropriate outlier exclusion techniques. However, our results are better than those obtained with other contactless techniques based on video [59], such as NIR cameras (MOD ± LOAs = 2.22 ± 8.0 breaths·min^−1^) or FIR cameras (MOD ± LOAs = 0.78 ± 4.24 breaths·min^−1^).

Regarding the HR estimation, we obtained comparable MAE and RMSE in all the trials when considering the HR computed with the multi-ROI approach. MAE is between 0.7 bpm and 6 bpm per each implemented post-processing technique, and RMSE is in the range of 1.2 bpm and 9.11 bpm. These values are comparable with those obtained in previous studies where the average HR values are computed from a single-ROI approach (RMSE of 4.97 bpm in [28] and RMSE of 6.92 bpm in [60]). To the best of our knowledge, no studies have performed continuous monitoring with similar approaches. Considering the influence of user–camera distance and lighting conditions, in line with results reported in previous studies, the values of MAE and RMSE increase with the distance [9] and when the light is off [12]. Comparing the HR results obtained with the classical single-ROI with those obtained with the multi-ROI approach, the latter are better per each implemented algorithm. When the SNR-based approach is used to estimate HR values from r-PPG signals extracted from each implemented algorithm, the values of MAE and RMSE are in the range 0.60 bpm and 6.71 bpm, and 1.27 bpm and 12.98 bpm, respectively, and are comparable with those obtained when the multi-ROI approach is used per each trial. In addition, from the Bland–Altman analysis, per each implemented post-processing technique, the values of LOAs are slightly high when compared to those obtained in the multi-ROI approach.

## 7. Conclusions

Despite the absence of contact with the subject, in this study, promising results are obtained in the context of the cardiorespiratory monitoring with a built-in smartphone’s camera, fostering the remote monitoring of the health status of a person and of individuals that require continuous monitoring. Our paper results demonstrated that both the proposed multi-ROI and SNR-based approaches could be reliable to estimate values of HR and *f_R_* with an update time of 1 s, providing a temporal trend of these values. However, some limitations have to be clarified, mainly related to the non-inclusion of subjects with different skin colors in the experimental trials and the non-investigation of motion artifacts’ influence in the estimation of cardiorespiratory parameters. These observations may be considered for further real-time applications, even in different scenarios (e.g., during sports activities, automotive field). Further investigations will be devoted to evaluating the influence of movements on the quality of r-PPG signals and the estimation of HR values. In addition, the recording and analysis of longer videos can be helpful to evaluate the performances of the proposed method in continuous monitoring of cardiorespiratory parameters, considering a more comprehensive range of HR and *f_R_*. Further efforts will be oriented to investigate the feasibility of estimating heart rate variability indexes in the time and frequency domains from signals retrieved with the SNR-based approach to better assess the physiological and psychological conditions of a person.

## Figures and Tables

**Table 1:** Experimental conditions: user–camera distance and lighting conditions. In the trial column, the number represents the user–camera distance in m, and Off/On is the absence or presence of light ring as additional light source, respectively.

Trial	User-Camera Distance [m]	Light Source	Illumination [lx]
0.5—Off	0.5	Natural Light	Median: 76.5; Range: 41–271
0.5—On	0.5	Light ring	Median: 180.5; Range: 148–288
1—Off	1.0	Natural Light	Median: 87.5; Range: 41–272
1—On	1.0	Light ring	Median: 149.5; Range: 114–289

**Table 2:** Mean Absolute Error (MAE) and Root Mean Square Error (RMSE) computed per each trial considering all the subjects. In bold are highlighted the best results.

Trial	MAE [Breaths·min^−1^]	RMSE [Breaths·min^−1^]
0.5—off	0.47	**1.74**
0.5—on	0.68	1.76
1—off	0.62	2.50
1—on	**0.42**	1.89

**Table 3:** MOD ± LOAs obtained in estimating HR from the three ROIs (single-ROI approach) and the average HR (obtained with the mean-ROI approach) per each implemented post-processing technique in the different ambient conditions. The best results are highlighted in bold.

		Light on, Light off		Light on, Light off	
r-PPG Post-Processing Techniques	ROI	d = 0.5 m	d = 1.0 m	d = 0.5 m	d = 1.0 m
		MOD ± LOAs [bpm]	MOD ± LOAs [bpm]	MOD ± LOAs [bpm]	MOD ± LOAs [bpm]
Green Channel	LCheek	−0.11 ± 4.01	−0.36 ± 8.74	−0.18 ± 6.33	−0.28 ± 7.24
	FHead	−0.07 ± 8.92	−0.20 ± 5.78	0.03 ± 6.15	−0.30 ± 8.65
	RCheek	−0.04 ± 4.45	−0.56 ± 7.81	−0.04 ± 3.79	−0.56 ± 8.15
	ROI_mean_	**−0.07 ± 4.06**	**−0.37 ± 4.91**	**−0.06 ± 3.65**	**−0.38 ± 5.22**
*modwtmra*	LCheek	0.27 ± 3.31	0.57 ± 6.29	0.20 ± 4.49	0.64 ± 5.49
	FHead	0.34 ± 4.10	0.88 ± 6.78	0.48 ± 4.59	0.74 ± 6.49
	RCheek	0.31 ± 5.00	0.31 ± 7.46	0.43 ± 5.47	0.19 ± 7.12
	ROI_mean_	**0.31 ± 3.03**	**0.59 ± 5.73**	**0.37 ± 3.95**	**0.52 ± 5.15**
ICA	LCheek	**−0.06 ± 3.32**	−0.04 ± 12.98	0.24 ± 9.20	−0.35 ± 9.70
	FHead	−0.008 ± 7.06	0.30 ± 7.62	**−0.10 ± 3.86**	0.39 ± 9.63
	RCheek	−0.03 ± 3.71	0.26 ± 11.38	0.09 ± 4.41	0.13 ± 11.14
	ROI_mean_	−0.03 ± 3.47	**0.17 ± 6.72**	0.08 ± 3.95	**0.06 ± 6.45**
PCA	LCheek	0.68 ± 16.67	1.15 ± 19.44	0.76 ± 18.51	1.06 ± 17.71
	FHead	0.54 ± 16.52	3.02 ± 19.92	1.40 ± 20.35	2.16 ± 16.33
	RCheek	0.43 ± 15.56	1.62 ± 22.36	1.19 ± 15.16	0.86 ± 22.69
	ROI_mean_	**0.55 ± 10.36**	**1.93 ± 12.57**	**1.12 ± 11.69**	**1.36 ± 11.50**
POS	LCheek	0.07 ± 3.63	0.23 ± 11.95	−0.07 ± 3.97	0.37 ± 11.83
	FHead	0.19 ± 4.83	0.65 ± 9.68	0.05 ± 2.89	0.79 ± 10.40
	RCheek	−0.03 ± 2.96	−0.06 ± 6.84	0.05 ± 2.91	−0.14 ± 6.85
	ROI_mean_	**0.07 ± 2.67**	**0.27 ± 6.29**	**0.009 ± 2.45**	**0.34 ± 6.36**
CHROM	LCheek	−0.32 ± 6.32	3.15 ± 19.41	0.62 ± 7.06	2.21 ± 19.62
	FHead	0.07 ± 5.81	3.38 ± 18.95	0.74 ± 12.47	2.71 ± 15.84
	RCheek	−0.17 ± 4.70	3.05 ± 19.20	1.02 ± 9.03	1.86 ± 18.11
	ROI_mean_	**−0.14 ± 3.71**	**3.19 ± 12.51**	**0.79 ± 6.20**	**2.26 ± 12.21**

**Table 4:** Mean Absolute Error (MAE) and Root Mean Square Error (RMSE) computed per each trial and each implemented post-processing technique considering the average HR. The best results are highlighted in bold.

Trial	MAE [bpm]						RMSE [bpm]					
	r-PPG Post-Processing Techniques						r-PPG Post-Processing Techniques					
	GC	*modwtmra*	ICA	PCA	POS	CHROM	GC	*modwtmra*	ICA	PCA	POS	CHROM
0.5—off	0.85	0.94	1.04	4.21	**0.69**	2.32	1.85	2.40	2.43	6.84	**1.20**	4.34
0.5—on	0.80	0.80	0.76	3.44	**0.65**	0.84	1.87	1.62	1.49	5.17	**1.30**	1.55
1—off	**1.63**	1.93	2.25	4.56	2.71	5.91	**3.07**	3.46	4.20	6.54	4.39	9.11
1—on	0.82	**0.74**	0.90	3.54	0.77	1.21	2.25	1.53	2.01	5.45	**1.43**	2.19

**Table 5:** MOD ± LOAs obtained in the estimation of HR from r-PPG signals with the SNR-based approach per each implemented post-processing technique in the different ambient conditions. In bold are highlighted the best results.

	Light On, Light Off		Light On, Light Off	
r-PPG Post-Processing Techniques	d = 0.5 m	d = 1.0 m	d = 0.5 m	d = 1.0 m
	MOD ± LOAs [bpm]	MOD ± LOAs [bpm]	MOD ± LOAs [bpm]	MOD ± LOAs [bpm]
Green Channel	−0.04 ± 3.31	**−0.38 ± 6.30**	**−0.07 ± 2.72**	**−0.35 ± 6.58**
*modwtmra*	0.31 ± 5.00	0.31 ± 7.46	0.43 ± 5.47	0.19 ± 7.12
ICA	0.22 ± 19.28	0.71 ± 13.39	−0.42 ± 10.80	1.35 ± 20.72
PCA	0.85 ± 16.39	0.57 ± 18.25	1.40 ± 13.63	0.02 ± 20.31
POS	**−0.03 ± 2.96**	−0.06 ± 6.84	0.05 ± 2.91	−0.14 ± 6.85
CHROM	−0.17 ± 4.70	3.05 ± 19.20	1.02 ± 9.03	1.86 ± 18.11

**Table 6:** Mean Absolute Error (MAE) and Root Mean Square Error (RMSE) in bpm computed for HR values estimated from the r-PPG signals with the SNR-based approach per each implemented post-processing technique. The best results are highlighted in bold.

Trial	MAE [bpm]						RMSE [bpm]					
	r-PPG Post-Processing Techniques						r-PPG Post-Processing Techniques					
	GC	*modwtmra*	ICA	PCA	POS	CHROM	GC	*modwtmra*	ICA	PCA	POS	CHROM
0.5—off	**0.60**	0.84	0.91	3.59	0.68	2.64	**1.27**	2.55	2.05	8.81	1.44	6.47
0.5—on	**0.73**	1.10	2.83	1.82	0.74	0.82	**1.49**	3.07	7.54	4.79	1.53	1.63
1—off	**1.77**	2.31	4.76	5.31	2.44	6.71	**4.40**	4.76	9.49	9.81	4.72	12.98
1—on	0.80	0.84	5.49	4.82	**0.78**	1.40	1.86	1.94	11.69	10.87	**1.49**	2.98

## Data Availability

The data presented in this study are available on request from the corresponding author. The data are not publicly available due to privacy restrictions.
